# Differences in Evolution of Epileptic Seizures and Topographical Distribution of Tissue Damage in Selected Limbic Structures Between Male and Female Rats Submitted to the Pilocarpine Model

**DOI:** 10.3389/fneur.2022.802587

**Published:** 2022-04-05

**Authors:** Daniel Matovu, Esper A. Cavalheiro

**Affiliations:** Neuroscience Laboratory, Department of Neurology and Neurosurgery, Escola Paulista de Medicina/UNIFESP, São Paulo, Brazil

**Keywords:** seizure patterns, pilocarpine model of epilepsy, male and female rats, olfactory bulb, amygdala, hippocampus

## Abstract

Epidemiological evidence shows that clinical features and comorbidities in temporal lobe epilepsy (TLE) may have different manifestations depending on the sex of patients. However, little is known about how sex-related mechanisms can interfere with the processes underlying the epileptic phenomenon. The findings of this study show that male rats with epilepsy in the pilocarpine model have longer-lasting and more severe epileptic seizures, while female rats have a higher frequency of epileptic seizures and a greater number of seizure clusters. Significant sex-linked pathological changes were also observed: epileptic brains of male and female rats showed differences in mass reduction of 41.8% in the amygdala and 18.2% in the olfactory bulb, while loss of neuronal cells was present in the hippocampus (12.3%), amygdala (18.1%), and olfactory bulb (7.5%). Another important sex-related finding was the changes in non-neuronal cells with increments for the hippocampus (36.1%), amygdala (14.7%), and olfactory bulb (37%). Taken together, our study suggests that these neuropathological changes may underlie the differences in the clinical features of epileptic seizures observed in male and female rats.

## Introduction

Epilepsy is a brain disorder with electroclinical characteristics presenting recurrent non-evoked seizures, often associated with significant psychological morbidity and complications (1, 2). Epidemiological evidence reporting on sex differences in epilepsy is surprisingly scanty (3). Studies show that epilepsy prevalence, mortality, and tonic–clonic seizures are higher in men than women, while specific epileptic syndromes are more prevalent in women (3, 4). As to psychiatric comorbidities associated with epilepsy, depression seems to be more frequent in women, while anxiety disorders are more common in men (5, 6).

Understanding mechanisms underlying sex-related differences in human epilepsy is important as they could contribute to better management of the clinical and therapeutic features observed in these patients. However, this kind of investigation is not always feasible in human studies, and experimental research can often provide useful information in the translational context of this pathology (7–9). Observations carried out by our group showed that male and female rats presented very similar characteristics in the period of status epilepticus (SE) induced by pilocarpine. However, important behavioral differences between these groups were verified in the chronic phase of the experimental model (10–13). For instance, female rats manifested disrupted maternal behavior including cannibalism of their pups, whereas male rats exhibited hyposexuality and were more aggressive. Other behavioral abnormalities found in mice and rats submitted to the pilocarpine model include anxiety (14, 15) and depressive-like behavior (16, 17). These findings may suggest that structural brain changes resulting from pilocarpine-induced SE are responsible for the emergence of spontaneous and recurrent seizures associated with behavioral changes according to the animal's sex (12). In this sense, understanding what happens during the epileptogenic process in these animals can help us to design insights into the pathophysiology underlying the differences in the manifestation of epilepsy between male and female rats.

Interestingly, behavioral disturbances like those observed in rats submitted to the pilocarpine model, such as disruption of maternal behavior in females and aggressiveness in males, are also observed in rats with olfactory bulbectomy (18–20). The rodent olfactory bulb is a crucial part of a neuronal circuit whose sensory functionality can determine the animal's immediate survival *via* activities such as foraging, predator recognition, social interaction, reproduction, and many other aspects of behavior (21). Neurons present in the olfactory bulb form several connections with various structures of the brain (22). Through efferent connections to the amygdala and hippocampus, the olfactory bulb sends information to limbic structures where the modulation and orchestration of emotional and behavioral responses occur (21). In addition, it is impressive to note the importance of olfactory stimuli for the relationship between rodent mothers and their pups. Indeed, neo neurogenesis and extensive reorganization of neuronal circuitry occur in the olfactory bulb and associated structures during pregnancy, parturition, and the puerperium of rats and mice (23–25).

This intriguing experimental evidence motivated us to study the evolution of epileptic seizures and the topographic distribution of tissue damage that occurs in male and female rats submitted to the pilocarpine model. Neuropathological changes in the olfactory bulb, amygdala, and hippocampus associated with the occurrence of recurrent seizures may reveal the direct involvement of a primary sensory pathway in the behavioral changes observed in the pilocarpine model of TLE. Specifically, we evaluated the developmental patterns of pilocarpine-induced epilepsy and the most striking features of recurrent epileptic seizures in male and female Wistar rats by comparing: the general characteristics of the acute phase; the duration of the latent period; the frequency, duration, and severity of each spontaneous seizure during the chronic period; and the degree of cellular damage in the olfactory bulb, amygdala, and hippocampus.

## Materials and Methods

### Ethical Statement

This study was approved by the Ethics Committee on Animal Use of the Federal University of São Paulo (CEUA / UNIFESP) under the reference number 8624210118. All experiments were following the international guidelines for animal care and conducting *in vivo* experiments on research animals (26).

### Animals and Experimental Groups

Thirty Wistar rats of ~60 days of age were used, consisting of male and female rats with an initial weight between 210 and 245 g, which were divided into 4 groups: control males (*n* = 5), control females (*n* = 5), males with epilepsy (*n* = 10), and females with epilepsy (*n* = 10). The animals were housed in groups of four per cage under controlled climatic conditions (12-h light–dark cycle, lights on between 7:00 a.m. and 7:00 p.m. and constant temperature (19–23°C), with free access to water and food.

### Vaginal Cytology

A non-invasive dropper was introduced into the vagina of Wistar rats to obtain the vaginal contents. The content obtained was analyzed under optical microscopy to determine the pattern of the estrous cycle of these animals. Such examination was performed at an interval of 8–10 h for 4 consecutive days (duration of the estrous cycle in female rats). Only female animals in the typical estrous phase were included in the study and submitted to pilocarpine administration (27).

### Induction of the Epilepsy Model by Pilocarpine

The pilocarpine model of epilepsy represents a suitable paradigm for studying the pathophysiological mechanisms of TLE and has been used in several studies (28). In summary, male and female Wistar rats were pre-treated with methyl scopolamine (Sigma Co.) at a dose of 1 mg/kg subcutaneously to inhibit the peripheral cholinergic effects of pilocarpine (12). Thirty minutes later, pilocarpine HCl (Sigma Co.) at a dose of 300 mg/kg was injected intraperitoneally with the aim of inducing long-standing SE (12, 29). After 5 h of SE, diazepam (Merck) at a dose of 10 mg/kg was administered subcutaneously to limit motor seizures (30). The surviving animals (7 males and 9 females) were fed a special fractionated diet until complete recovery, which ends the acute period of the model. After this period, the animals were transferred to video surveillance. Control male and female rats were injected with methyl scopolamine (1 mg/kg, s.c.) followed 30 min later by a saline injection (1 ml/kg, i.p.) as a substitute for pilocarpine.

### Monitoring of Epileptic Seizures

A high-definition video system (Intelbras VT 4 S 120 HD) was used to detect the first spontaneous epileptic seizure, which inaugurated the chronic period of the experimental model and, later, to continuously monitor (24 h/day) the recurrent seizures typical of this model (31). The following parameters were recorded: number, duration, and severity (by Racine scale) of each spontaneous seizure. The distribution of seizures per 24 h was recorded for the light (7 a.m.−6.59 p.m.) and dark (7 p.m.−6.59 a.m.) periods. The severity of each seizure was classified according to the scale proposed by Racine for amygdala kindling (32): (1). orofacial automatisms; (2). clonus of the head; (3). forelimb clonus; (4). forelimb clonus plus rearing; and 5. generalized clonic movements with alternating rearing and falling. The occurrence of seizure cluster (33) was also recorded.

### Isotropic Fractionator Method

This method is used to quantify the number of neuronal and non-neuronal cells throughout the brain or in selected brain structures. This technique does not need any special software but follows similar principles used in stereological analysis (34). Briefly, at the end of the 3-month observation period, all animals in each group (male and female controls and males and females with epilepsy) were anesthetized with sodium thiopental (80 mg/kg, s.c.) and perfused transcardially with buffered saline solution, phosphate (0.01 M; pH 7.4), followed by 4% paraformaldehyde. Their brains were carefully removed and fixed in 4% paraformaldehyde for 24 h. Under a Leica stereomicroscope, brain slices (1 mm, Kent) were fixed through crushed ice, and with the aid of consistent anatomical landmarks, it was possible to obtain the tissue of the 3 target structures of this work: olfactory bulb, amygdala, and hippocampal formation. The olfactory bulbs were separated by cutting near the frontal poles. The hippocampal formation was carefully dissected from each hemisphere, detaching it from the striatum and cortical tissue. The amigdalae were identified laterally to the hypothalamus and to the ventral portion of the rhinal sulcus, allowing the isolation of the amygdala composed of its basolateral and centromedial portions (35). Each brain structure was mechanically dissociated in saline solution with 1% Triton X-100 and transformed into an isotropic suspension of isolated nuclei, kept homogeneous by shaking. The total number of cells was estimated by determining the number of nuclei in small aliquots stained with the fluorescent DNA marker 4'-6-diamidino-2-phenylindole dihydrochloride (DAPI, 1:1000; Sigma-Aldrich, D9542) using a microscope Zeiss Axiovert 100 with 40x objective and by using hemocytometer for quantification (Neubauer chamber, Loptik Labor). To determine the number of neuronal and non-neuronal cells, samples were incubated with the primary antibody against the specific neuronal nuclear protein (NeuN, 1:100; Abcam, ab104225) at 4°C overnight and subsequently with secondary antibody conjugated with AlexaFluor ® 488 (Abcam; ab150077) diluted in PBS (1:200) and 10% normal goat serum (Vector labs, S-1000-20) for 2 h. The neuronal fraction in each sample was estimated by counting NeuN-stained nuclei in at least 500 DAPI-stained nuclei, and the number of non-neuronal nuclei was obtained by subtraction (36).

### Statistical Analysis

The Shapiro–Wilk test was performed to verify data normality using Graph Pad Prism version 8.4.2 (Graph Pad Software, La Jolla California USA). The unpaired t-test was used to analyze the duration of the latent period in animals treated with pilocarpine. Two-way ANOVA (repeated measures) and Sidak's *post-hoc* test were performed for seizure-dependent variables. Independent two-way ANOVA and Tukey's *post-hoc* test were performed on dependent variables: mass, number of neurons, and non-neuronal cells of brain structures. Data were presented as mean and standard deviation with a significance level of 0.05.

## Results

### The Pilocarpine Epilepsy Model in Male and Female Rats

The systemic administration of pilocarpine in male and female rats promotes sequential behavioral and electrographic changes that are divided into three distinct periods: (a) an acute period that built up progressively into a limbic SE and that lasts 24 h, (b) a latent or silent period with a progressive normalization of EEG and behavior which varies from 4 to 44 days, and (c) a chronic period with spontaneous recurrent seizures (SRSs).

In the acute period, no marked difference was observed between male and female rats. SE had its onset between 15 and 37.8 min after pilocarpine administration and the most predominant behavioral manifestation observed was the presence of limbic seizures characterized by continuous orofacial automatisms and chewing, salivation, head nodding, and forelimb cloning. Every 20–40 min, these changes progressed to generalized seizures with an average duration of 30 s to 1 min, at the end of which there was a transient decrease in behavior before returning to the predominant limbic seizures already described. Male and female animals treated with pilocarpine received diazepam 5 h after the start of SE to reduce the strong behavioral manifestations observed during this period. Even so, 3 male and 1 female rat died in this acute phase of the model. The behavior of all survivors was similar to that of control animals 24–48 h after pilocarpine injection, which signaled the end of the acute period and the beginning of the latent period of the experimental model.

After a period of ~2 weeks, the surviving animals presented the first spontaneous seizure, ending the latent period and inaugurating the chronic period of the model whose main characteristic is the occurrence of spontaneous and recurrent seizures. In the present study, the duration of the latent period was not different between male (15.1 ± 2.7 days) and female (14.6 ± 6.6 days) rats. The spontaneous seizures observed thereafter resembled those of stage 3 or higher on the Racine scale and recurred variably between male and female rats.

**Figure 1:**
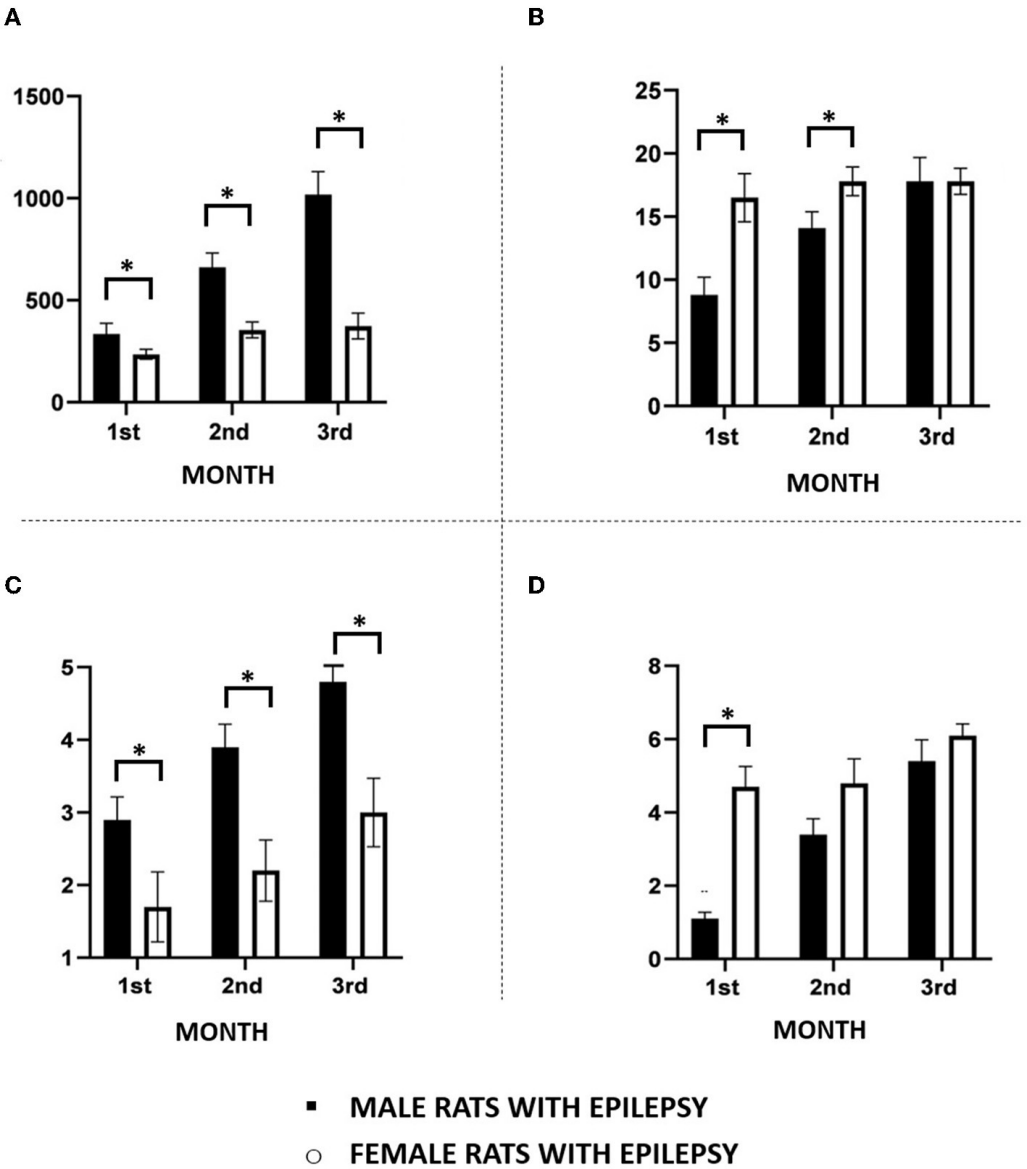
Main features of spontaneous recurrent seizures observed in male and female rats during the chronic period of the pilocarpine model of epilepsy monitored continuously 24 h for a period of 3 months. **(A)** Seizure duration; **(B)** Seizure frequency; **(C)** Seizure severity; **(D)** Seizure clusters. Data are expressed as mean ± SD. Data from male and female rats were compared using two-way ANOVA (multiple measures) followed by Sidak's *post-hoc* test. *p < 0.05.

As can be seen in Figure 1, the duration of spontaneous epileptic seizures analyzed in the 3 months of the study was consistently longer in male rats than in female rats. On the other hand, females had more spontaneous seizures than male rats in the first 2 months of observation (Figure 1B). In the third month, the difference between the number of spontaneous seizures in male and female rats was no longer observed. These spontaneous seizures were significantly more severe in male rats than in females during all the 3 months of the study. As can be seen in Figure 1C, Grade 4 and 5 seizures (Racine scale) were frequent in male animals, but rarely observed in female animals. Regarding the cluster of seizures, we observed that its occurrence was higher in female rats than in males only in the first month of observation (Figure 1D).

**Figure 2:**
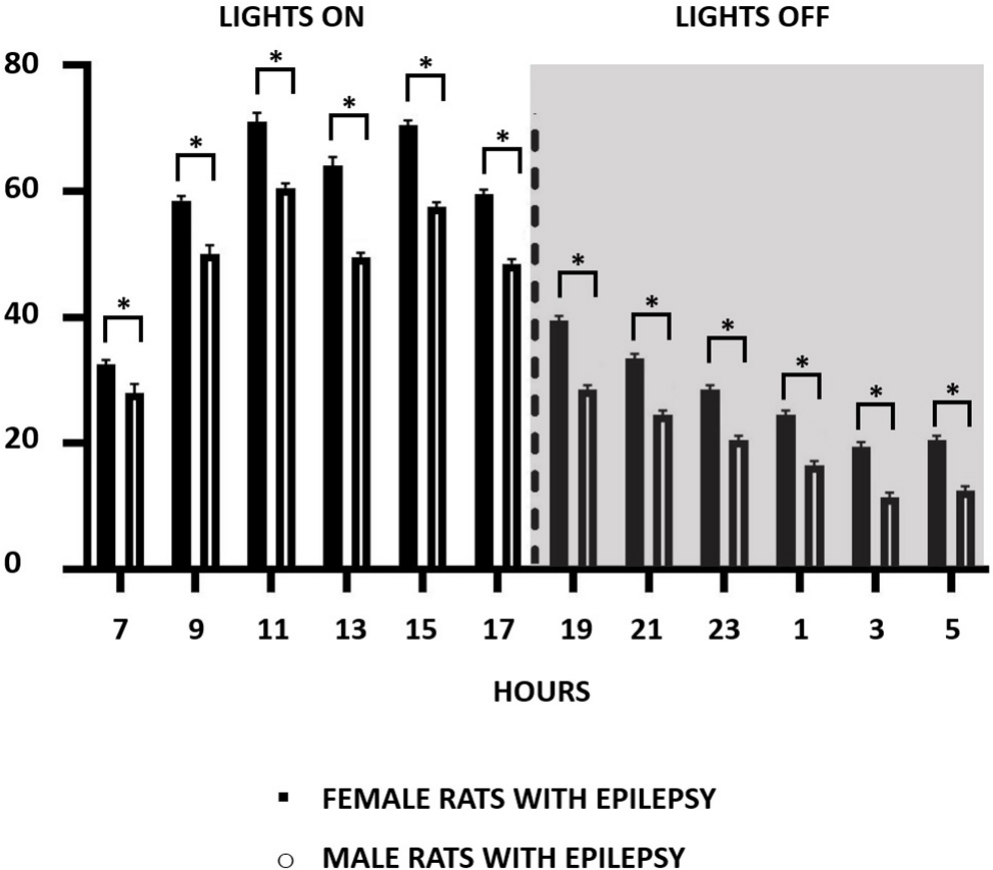
Number of recurrent seizure distribution in a 2-h pattern for 24-h recordings over a period of 3 months during the light (07:00–18:59 h) and dark (19:00–06:59 h) cycle in both male and female epileptic rats. Data are expressed as mean ± SD. *p < 0.05.

Considering that the occurrence of epileptic seizures varies according to the 24-h light/dark cycle, we compared their occurrence in male and female rats throughout the study. In this way, and as can be seen in Figure 2, the effect of light and dark in the 24-h period was similar in male and female rats, with more seizures in the light than in the dark period. An interesting observation in this type of study was to verify that the greater number of spontaneous seizures in females, as seen in Figure 1B, does not occur in a specific period of the day or night, but that this increase is evenly distributed throughout the period of 24 h.

### Structural Changes in the Brain of Male and Female Rats Submitted to the Pilocarpine Model of Epilepsy

**Figure 3:**
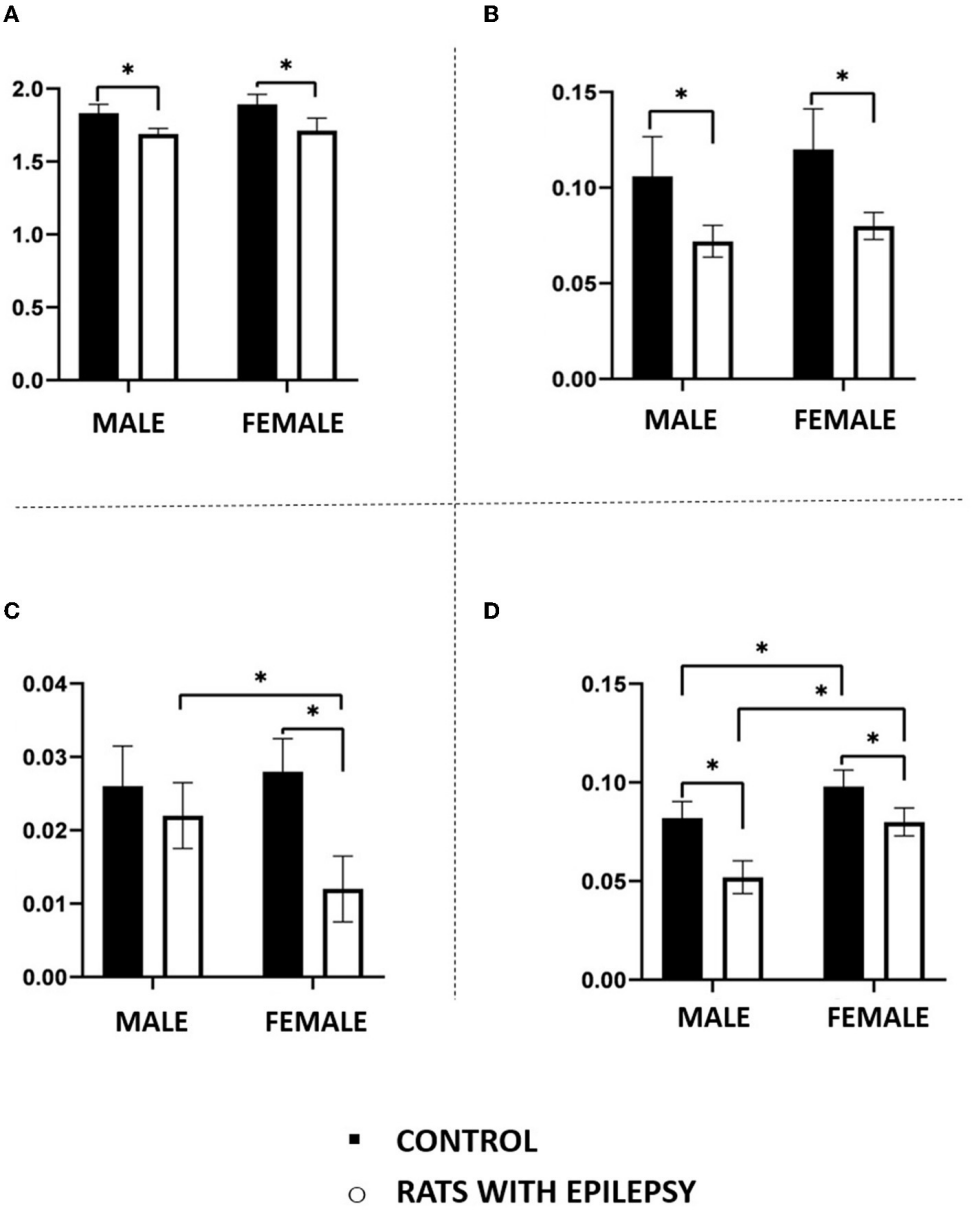
Mass (in grams) in the total brain **(A)**, hippocampal formation **(B)**, amygdala **(C)**, and olfactory bulb **(D)** obtained from male and female rats submitted to the pilocarpine model of epilepsy in relation to that of the respective controls. Data are expressed as mean ± SD. Data from rats with epilepsy were compared with those of their respective controls using two-way ANOVA followed by Tukey's *post-hoc* test. *p < 0.05.

The mass (in grams) of the total brain and the three structures under study, i.e., olfactory bulb, amygdala, and hippocampal formation, showed interesting and significant differences between male and female rats with spontaneous and recurrent epileptic seizures for 3 months. As expected, the brain weight of animals with epilepsy was lower than that observed in their controls, but not different between male and female animals with epilepsy (Figure 3A). This observation was also replicated for the hippocampal formation (Figure 3B).

However, even confirming that the weight of the amygdala and the olfactory bulb of the animals with epilepsy was lower than that observed in the controls, there were differences related to sex not previously anticipated. As can be seen in Figures 3C,D, female rats with epilepsy had amygdala with lower weight than males with epilepsy, while the latter had olfactory bulbs with lower weight than females with epilepsy.

**Figure 4:**
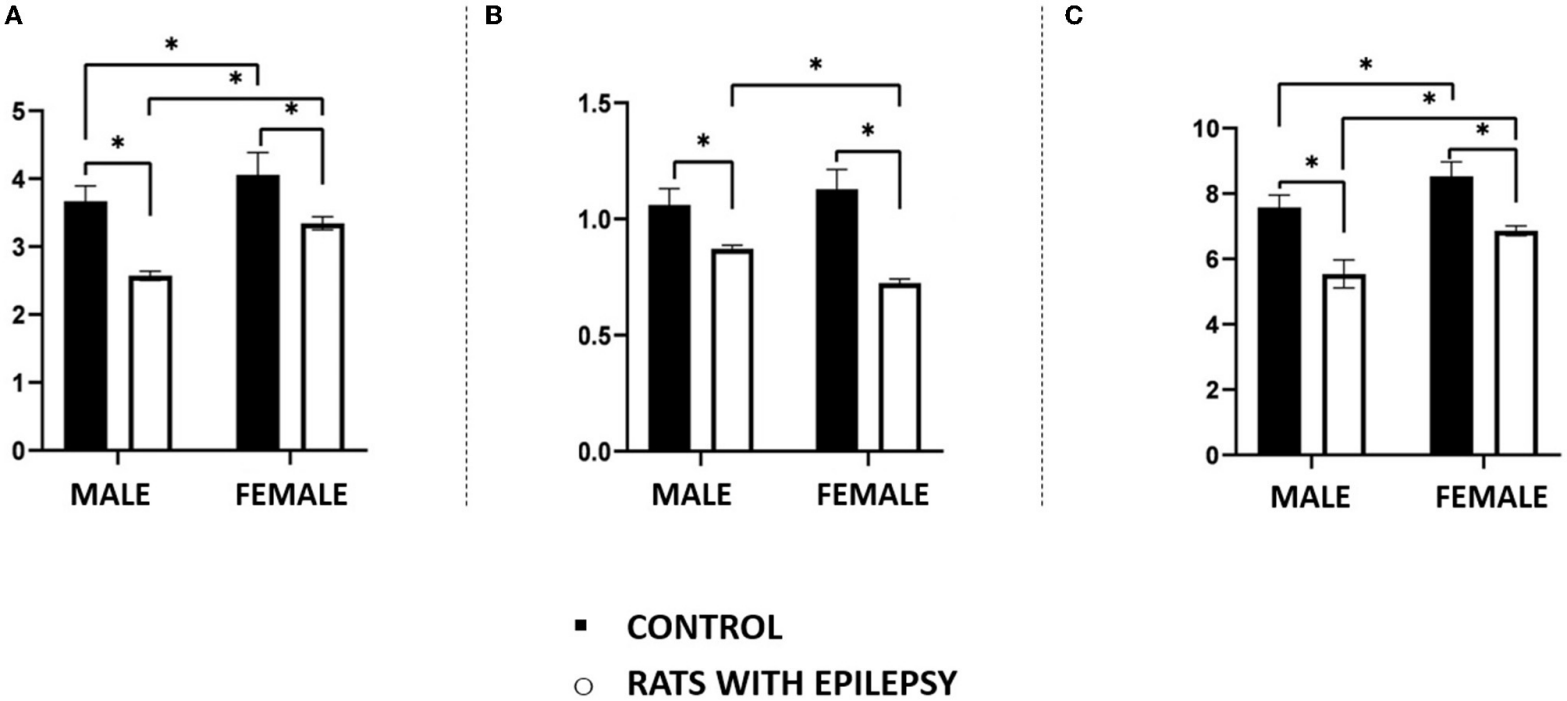
Number (in millions) of neurons in the hippocampus **(A)**, amygdala **(B)**, and olfactory bulb **(C)** of male and female rats submitted to the pilocarpine model of epilepsy compared to that observed in respective controls. Data are expressed as mean ± SD. Data from rats with epilepsy were compared with those of their respective controls using two-way ANOVA followed by Tukey's *post-hoc* test. *p < 0.05.

**Figure 5:**
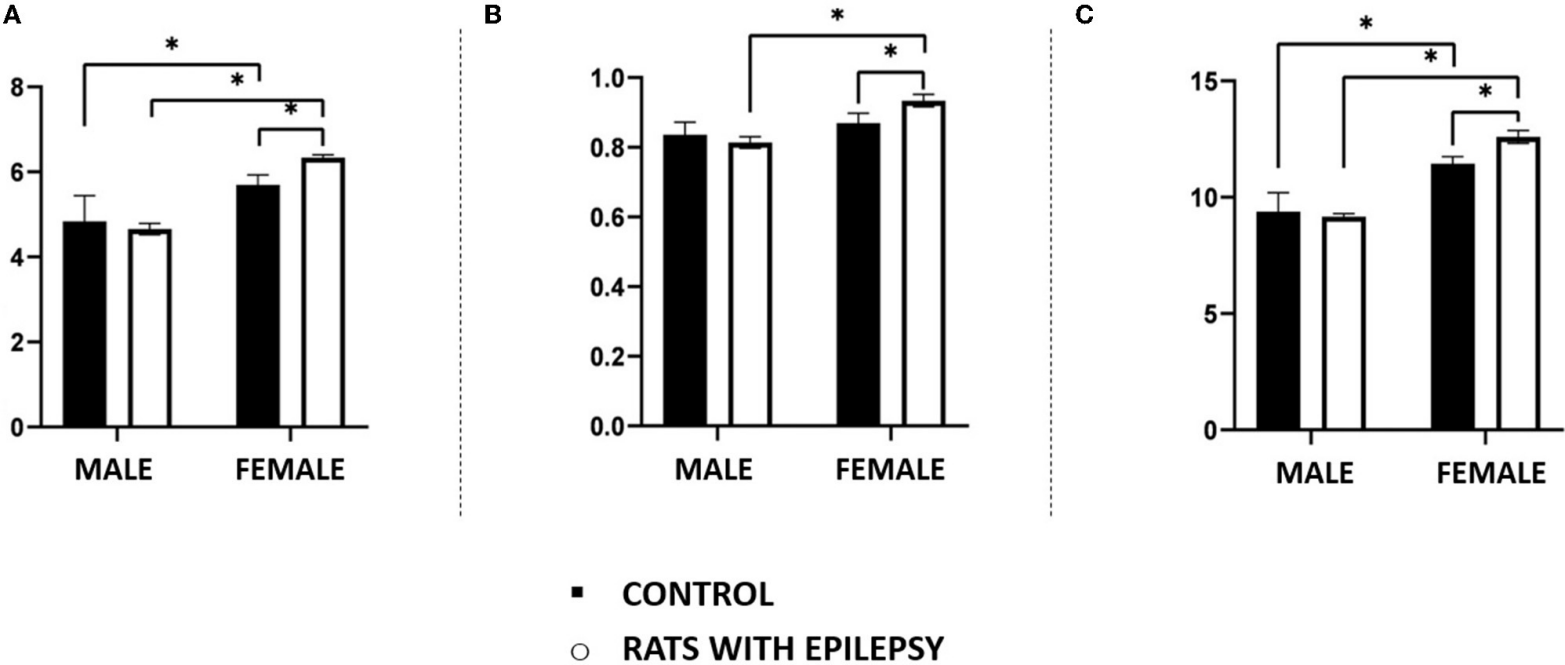
Number (in millions) of non-neuronal cells in the hippocampus **(A)**, amygdala **(B)**, and olfactory bulb **(C)** of male and female rats submitted to the pilocarpine model of epilepsy compared to that observed in respective controls. Data are expressed as mean ± SD. Data from rats with epilepsy were compared with those of their respective controls using two-way ANOVA followed by Tukey's *post-hoc* test. *p < 0.05.

Neuronal and non-neuronal cell counts in these three structures also yielded interesting observations (Figures 4, 5). The number of neurons found in the hippocampal formation, amygdala, and olfactory bulb of male and female rats with epilepsy was lower than those of the respective controls. However, the neuronal loss that occurred in the hippocampal formation and the olfactory bulb was more significant in male rats than in female rats with epilepsy. On the other hand, neuronal loss in the amygdala was greater in female rats than in male rats with epilepsy (Figure 4). Regarding the non-neuronal cellular component, we observed that its number in male rats with epilepsy was similar to that in control animals. In female rats with epilepsy, the set of non-neuronal cells was more numerous than that observed in their controls, which allows us to say that there was a significant difference in the number of non-neural cells between male rats and female rats with epilepsy (Figure 5).

## Discussion

The present study provides two major new findings in the rat epileptic brain. First, there are significant differences in the patterns of spontaneous recurrent seizures between male and female rats subjected to the pilocarpine model of epilepsy. Second, the topographical distribution of neuronal and non-neuronal cell death differs in the brains of male and female animals with epilepsy. These findings provide a possible explanation for the limbic circuit dysfunction and the occurrence of behavioral disturbances seen in the pilocarpine model of epilepsy. There is scanty data on patterns of epileptic seizures in rats; most of this information has been adapted for seizures observed during SE that are not suitable for comparison with spontaneous seizures that occur in the chronic phase of the model (37–39). Progressive maturation of epileptic seizures has been documented in male animals during the induction of several models of epilepsy and in human studies of people with TLE (40–44), although these studies do not fully show the wide range of discrepancies in seizure variables that our study demonstrates. However, the exact mechanisms by which dysfunctional microcircuits can trigger, propagate, or stop epileptic seizures of the most varied patterns remain undefined (41).

The results of our study present significant observations on the pattern of epileptic seizures that occur in male animals in relation to that observed in female animals. Male rats with epilepsy had epileptic seizures of greater severity and longer duration, while female rats with epilepsy had more frequent seizures, in addition to the presence of cluster of seizures in the first 2 months of observation. Brain morphological alterations underlying the epileptic process were also influenced by the sex of the animal. The brains of male and female rats with epilepsy showed differences in the reduction of amygdala (41.8%) and olfactory bulb (18.2%) mass. Neuronal and non-neuronal cell counts were also shown to be differently influenced by sex in the formation of the hippocampus, amygdala, and olfactory bulb.

In the pilocarpine model of epilepsy, as in similar models, the occurrence of long-term SE is the initial precipitating factor for epileptogenesis and the later development of epilepsy (2). The present study used pilocarpine-induced SE for 5 h. The rationale for this time was based on a previous study showing that brain damage induced by SE varied according to the duration of SE (30), and that animals submitted to 5 h of SE developed epilepsy in the long run similarly to those with SE lasting 8 h or more, although in the latter, mortality was 4–5 times higher than that of the group with 5 h of SE.

Previous work by our group and other laboratories showed that brain injuries in the pilocarpine model are not restricted to the three structures studied in this study (29, 45), which seem to depend on the duration of SE. Among these structures are the striatum, some thalamic, and brainstem nuclei. In human cases of TLE, thalamic alterations are already well described in the literature (46–48). However, in this study, we decided to focus on 3 limbic structures directly involved in the pathophysiology of TLE. Future experiments focused on other brain structures linked to TLE could indicate whether they are also influenced by sex. Studies in people with epilepsy (49–52) are also beginning to point to structural changes related to the sex of patients. In comparison to gender-matched controls, male patients presented gray matter volume reductions in thalamus and frontal gyri, while female exhibited reductions in temporal areas, thalamus, and cerebellum (53).

Pilocarpine-induced neuronal death in the limbic circuitry follows a cascade of events ranging from activation of muscarinic receptors to hyperactivation of AMPA and NMDA receptors, culminating in a significant increase in calcium permeability that leads to cell death. Insights into the development of hyperexcitable circuits from glutamatergic activation have been gained in kindling studies (54). For example, kindling of the olfactory bulb facilitates the propagation of seizure activity through extensive connections with the amygdala and hippocampus, leading to permanent structural changes in these structures (55). Thus, it is possible that the occurrence of pilocarpine-induced long-lasting SE facilitates the creation of a hyperexcitable limbic circuitry that includes connections with the olfactory bulb.

The different distribution of neuronal and non-neuronal cell death in the hippocampus, amygdala, and olfactory bulb between male and female rats with epilepsy can be attributed to the differential distribution of sex hormone receptors in these same structures, to the interneuron circuitry formed throughout life, and also to other factors such as the circadian cycle. It is well known that female rats express higher density and differential distribution of estrogen receptors in the limbic structures compared to male rats, as well as the influence of estrous cycle and circadian pattern on the number and function of these receptors (56).

The hormonal environment plays a significant role in sex-linked differences and related brain damage. Male rats undergoing lithium-pilocarpine-induced SE that received subcutaneous injections of 17β estradiol for 4 days exhibited a higher hippocampal neuron damage score compared to ovariectomized rats (57). These findings are in line with the increased loss of hippocampal neurons in male rats compared to female rats with epilepsy. Furthermore, our laboratory has previously demonstrated that female rats with epilepsy in the pilocarpine model have increased estradiol release and reduced progesterone levels (12). During the estrous cycle, female rats with epilepsy are susceptible to progesterone withdrawal, predisposing to increased seizure frequency and decreased allopregnanolone levels (58).

The mechanisms by which these two hormones facilitate or inhibit epileptic activity in limbic circuits are not fully understood (12). In summary, estradiol is a bidirectional ligand that exerts its proconvulsant effects through NMDA receptor and anticonvulsant through neuropeptide Y (59, 60). Emerging evidence has shown that progesterone is also a bidirectional modulator of neuronal excitability. Progesterone exerts its proconvulsant effect through regulation of AMPA receptor expression, facilitating enhanced glutamatergic transmission (61). In addition, progesterone mediates its anticonvulsant action through allopregnanolone (62), a metabolite of progesterone, which can also be synthesized in the body from cholesterol. Its anticonvulsant action is mainly due to its modulating action on central GABAergic receptors. Progesterone played an important role in controlling neuronal and non-neuronal cell death, neuroinflammation, and neurogenesis in rats with epilepsy (63). Testosterone, when converted to estradiol, shows proconvulsant action, and studies have shown that testosterone can induce intense tonic–clonic seizures in male rats with epilepsy (64, 65).

Insights from human studies of olfactory function in TLE have shown reduced size and function of the olfactory bulb for odor discrimination and identification in these patients (66). These findings agree with the high frequency of seizures observed in female rats with epilepsy and reduced olfactory bulb mass in pilocarpine-treated animals.

The isotropic cellular findings revealed that female rats with epilepsy showed a significant increase in the number of non-neuronal cells in the olfactory bulb, amygdala, and hippocampus when compared to male rats with epilepsy. Recent publications have shown that glial cell activation and behavior may differ depending on the sex of the animal (67, 68). Glial activation along with the activation of inflammatory cytokines in response to brain damage of different etiologies appears to depend on the presence of gonadal hormones (67, 69). Under these conditions, estradiol appears to have a protective and/or regenerative effect on glial cells more predominantly in female animals than in male animals (67, 69). Although intracellular mechanisms are not well understood, these findings agree with our study of a sustained increase in non-neuronal cells in limbic structures observed in female rats with epilepsy. In line with our results, GFAP positive astrocyte cell loss in the hippocampus has been reported in male rats with epilepsy 4 weeks after SE (70).

Together, both sex differences and the broad mechanistic effects of gonadal hormones on limbic excitability and vulnerability to epileptic seizures can be influenced by several factors, including dimorphic disparities in epileptogenic brain regions that trigger or stop seizures, intrinsic connectivity of the brain, distribution of receptors, and signaling pathways (71). These changes may contribute to differences in patterns of recurrent seizures, including frequency, duration, clustering, and seizure severity.

It is important to point out that during this study, the cell quantification method used here still did not allow the differentiation of non-neuronal cells. Now, this possibility already exists (personal communication from Professor Roberto Lent from the Federal University of Rio de Janeiro, Brazil), and we are organizing to start the experiments that will allow this differentiation in animals with epilepsy by pilocarpine. Another limitation of our study concerns the simultaneous recording of the EEG, which allows an immediate correlation with the behavioral findings, which is extremely important for the study of epilepsy. This was not possible due to the high risk of local infection through electrodes implanted for a long period, which could alter the results of the cell count.

Differential neuronal and non-neuronal cell death between male and female rats with epilepsy occurred in structures linked to relevant neuronal circuits associated with the organization and emission of behaviors and which, at the same time, participate in a sensory system that, predominantly for rodents, represents a window to the outside environment. Recurrent and long-lasting epileptic seizures promote the gradual loss of neurons (31). For example, studies in experimental epilepsy have shown important alterations in the GABAergic/parvalbumin circuitry of the olfactory bulb (72), the basolateral amygdala (54), and the hippocampus (73). It has also been observed that the CA1 region of the ventral hippocampus and the basolateral amygdala strongly depend on olfactory stimuli for the modulation of fear (74), mood, and anxiety, and these regions are precisely those that also exhibit the greatest degree of neuronal loss as a result of epileptic seizures (54, 75). Furthermore, these regions, associated with the prefrontal cortex and hypothalamus, also participate in the anxiety circuit (76, 77). Taken together, our data seem to indicate that in TLE olfactory glutamatergic dysfunction can induce hyperexcitability in regions of the amygdala and hippocampus, increasing seizure frequency and severity and altering the function of circuits responsible for controlling animal behavior.

Our results suggest that male and female rats with epilepsy differ in some central aspects of the experimental model, namely, in the frequency, duration, and severity of epileptic seizures, and in the loss of neuronal and non-neuronal cells in the olfactory bulb, amygdala, and hippocampus. These findings seem to indicate that sex-linked differences as well as spontaneous and recurrent epileptic seizures underlie differences in cell death patterns in structures linked to the limbic system and, thus, contribute to the occurrence of behavioral disturbances in this experimental model. Finally, this study was carried out in a special group of animals (Wistar rats) *via* a specific experimental model (pilocarpine epilepsy model) whose results need to be interpreted with caution when extrapolating to the human condition.

## Data Availability Statement

The data sets presented in this study can be found in https://repositorio.unifesp.br/xmlui/handle/11600/62173.

## Ethics Statement

The animal study was reviewed and approved by Ethics Committee on Animal Use of the Federal University of São Paulo (CEUA / UNIFESP) under the reference number 8624210118.

## Author Contributions

DM and EC designed the research, conducted the isotropic fractionator and neuronal quantification experiments, analyzed the data, and wrote the paper. DM performed the pilocarpine epilepsy experiments. Both authors approved the manuscript.

## Funding

This work was supported by a research grant from CNPq, Brazil (to EC) and an Institutional research grant to National Institute of Translational Neuroscience (MCTIC, CNPq, and FAPERJ of Brazil). DM was fellow from CAPES (Brazil) – Finance Code 001.

## Conflict of Interest

The authors declare that the research was conducted in the absence of any commercial or financial relationships that could be construed as a potential conflictof interest.

## Publisher's Note

All claims expressed in this article are solely those of the authors and do not necessarily represent those of their affiliated organizations, or those of the publisher, the editors and the reviewers. Any product that may be evaluated in this article, or claim that may be made by its manufacturer, is not guaranteed or endorsed by the publisher.
